# *O* ‐Acetyl‐Serine Supplementation Enhances Insulin Secretion and Improves Postprandial Glycaemia in Lean and Prediabetic Mice

**DOI:** 10.1096/fj.202502925R

**Published:** 2026-02-20

**Authors:** Clara Benatar, Xufei Zhang, Ines Haddam, Sandy Ribes, Sophie Bobet, Magali Monnoye, Elodie Lamy, Stanislas Grassin‐Delyle, Vincent Juillard, Séverine Layec, Christine Delorme, Véronique Douard

**Affiliations:** ^1^ Université Paris‐Saclay INRAE, AgroParisTech, MICALIS Institute Jouy‐en‐Josas France; ^2^ Département de Biotechnologie de la Santé, Infection et Inflammation (2I) U1173, UFR Simone Veil—Santé, Université Paris‐Saclay, UVSQ, INSERM Montigny le Bretonneux France; ^3^ Hôpital Foch Exhalomics Suresnes France

**Keywords:** GLP‐1, glycaemia, insulin, *O*‐acetyl‐serine, prediabetes

## Abstract

Type 2 Diabetes (T2D), often preceded by reversible prediabetes, poses a major health challenge. Targeting early glucose dysregulation is a promising preventive strategy. Amino acids and their derivatives are emerging as key regulators of glucose homeostasis. Here, we investigate the role of *O*‐acetyl‐serine (OAS), a serine‐derived metabolite produced by plants and microbes, including those of the gut microbiota, in glycaemia regulation. We developed a targeted method to quantify OAS in plasma and tissues, showing that it enters circulation and transiently accumulates in the pancreas. In vivo, OAS specifically and dose‐dependently improves postprandial glycaemia in lean mice. Mechanistically, OAS acts as a glucose‐dependent insulin secretagogue: a single oral dose increased pancreatic OAS levels by ~100‐fold and amplified glucose‐stimulated insulin secretion by 3.7‐fold, without affecting basal insulin levels. In vitro, OAS enhanced insulin secretion in both INS‐1 cells and rat islets, confirming a direct effect on pancreatic β‐cells. OAS also increased GLP‐1, but not GIP, levels at baseline and after glucose challenge. However, in vivo treatment with the GLP‐1 receptor antagonist exendin (9–39) revealed that GLP‐1 signaling only partially mediates OAS's effects, consistent with OAS direct action on insulin secretion. Finally, OAS restored glucose tolerance in a prediabetic mouse model induced by high‐fat diet, without altering insulin sensitivity. This improvement was associated with increased postprandial insulin and GLP‐1 levels. Together, these findings identify OAS as a glucose‐dependent insulin secretagogue with therapeutic potential to enhance insulin secretion and prevent progression from prediabetes to T2D.

## Graphical abstract

Following oral administration, *O*‐acetyl‐serine (OAS) enters the circulation and transiently accumulates in the pancreas, where it stimulates insulin secretion in a glucose‐dependent manner. OAS also stimulates GLP‐1 secretion, which may partially contribute to its insulin‐secretion–enhancing effects. Together, these actions lead to improved postprandial glucose control and restoration of glucose tolerance in prediabetic mice.

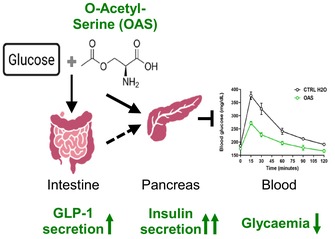

**Abbreviations**
AUC: area under the curve
BW: body weight
GIP: glucose‐dependent insulinotropic polypeptide
GLP‐1: glucagon‐like peptide‐1
HFD: high‐fat diet
HFHS: high‐fat high‐sugar
IPGTT: intraperitoneal glucose tolerance test
ITT: insulin tolerance test
NAS: N‐acetyl‐serine
NC: normal chow
OAS: O‐acetyl‐serine
OGTT: oral glucose tolerance test
T2D: type 2 diabetes

## 1 Introduction

Type 2 diabetes (T2D) is characterized by impaired glucose tolerance, leading to chronic hyperglycaemia related to changes in insulin secretion, defects in insulin sensitivity, or both [1]. Globally, 529 million people have T2D, with projections reaching 1.31 billion by 2050 [2]. Prediabetes, a reversible stage that precedes T2D, features blood glucose levels above normal but below T2D threshold. It is a significant risk factor for T2D, as nearly 70% of prediabetic individuals develop T2D during their lifetime [3, 4]. Early detection and management of prediabetes can prevent T2D, but lifestyle changes alone are only partially effective [5], indicating deeper metabolic mechanisms in glucose regulation.

Dietary amino acids, derived from protein degradation and those produced through the metabolic activities of the gut microbiota, as well as amino acid transporters, have long been recognized as key players in insulin signaling [6, 7, 8]. Advances in molecular profiling have expanded our understanding of diabetes pathophysiology and confirmed the importance of specific amino acids in predicting prediabetes and T2D outcomes [9, 10, 11, 12, 13, 14]. Metabolomics studies reported elevated circulating levels of branched‐chain amino acids (BCAA) (i.e., leucine, isoleucine and valine), aromatic amino acids (i.e., phenylalanine and tyrosine), as well as some essential amino acids (i.e., lysine and tryptophan) and non‐essential amino acids (i.e., glutamate, glutamine and aspartate) in T2D patients or those at higher risk of T2D [9, 12, 15, 16, 17, 18, 19, 20, 21, 22]. Conversely, serum levels of non‐essential amino acids like threonine, glycine, alanine, and serine, were reduced in diabetic individuals [18, 23, 24, 25, 26] and in T2D mouse models [27], suggesting potential protective effects, supported by improvements in T2D parameters in some amino acid supplementation studies [28, 29, 30]. Additionally, post‐translational modifications of proteins, such as acetylation, glycosylation, sulfation, formylation, and methylation‐phosphorylation, can alter the properties of amino acid residues [31], potentially affecting the metabolism and signaling functions of the resulting amino acid derivatives [32]. Interestingly, increased glycosylation, sulfation, acetylation, and carbamoylation of amino acids have been reported in the tissues and plasma of diabetic rodents and humans [33, 34, 35], suggesting a potential role of these modified amino acids in diabetes pathophysiology. Notably, 3‐methyl‐2‐oxovalerate, an isoleucine derivative, correlates more strongly with T2D and impaired fasting glucose than isoleucine itself, while *N*‐acetyl‐glycine and dimethyl‐arginine negatively correlate with T2D [36]. Other modified amino acids may also influence T2D, but their identity and specific roles remain largely unknown. Among these, *O*‐acetyl‐serine (OAS) is of particular interest given L‐serine's role in glycaemia regulation and T2D. OAS is a non‐proteinogenic‐amino acid synthetized by serine acetylation and is a central metabolite that links serine metabolism to sulfur assimilation, playing a crucial role in the biosynthesis of cysteine and the regulation of sulfur metabolism in plants, bacteria, archaea and fungi [37]. In these organisms, OAS acts both as a metabolic precursor and as a signaling molecule that regulates gene expression and enzyme activity in response to sulfur availability. It is chemically sensitive to pH, and can be converted into *N*‐acetyl‐serine (NAS) and/or reverted to serine through the loss of its acetyl group. Despite the inability of human cells to synthesize OAS, it has been detected, albeit in very few publications, in plasma (8 μM) and urine (247 μM) of healthy individuals [38, 39], likely originating from dietary vegetables [40, 41] and/or the metabolic activities of the gut microbiome [42, 43]. Notably, its plasmatic levels are comparable to those of cysteine, methionine and aspartate in human [39]. Nevertheless, little is known about OAS metabolism or its potential roles in eukaryotes outside of plants. In the current study, we investigated OAS's impact on normoglycaemic and prediabetic mice. We found that OAS improves postprandial glycaemia in both normoglycaemic and prediabetic mice. In addition, we demonstrated that OAS stimulates the secretion of GLP‐1 (Glucagon‐like peptide‐1) in intestinal cells and insulin in pancreatic β‐cells. These findings suggest that OAS regulates glycaemia by activating both GLP‐1 and insulin secretion.

## 2 Materials and Methods

### 2.1 Animals and Experimental Design

The animal experimental protocols were approved by the local ethics committee (COMETHEA, Jouy‐en‐Josas, France, APAFIS #38185‐2022080812185929 and APAFIS #38406‐2022090910117985). Cohorts of 8‐ to 10‐week‐old C57BL/6J male mice (Janvier Labs, Le Genest‐Saint‐Isle, France) were used. Mice were housed in groups of 2–3 per cage with a 12‐h daylight cycle. Normoglycaemic lean mice were fed a standard normal chow (NC) diet (Sniff, Soest, Germany) ad libitum. *O*‐acetyl‐L‐serine hydrochloride (OAS), L‐Serine, and *N*‐acetyl‐L‐serine (NAS) (all from Sigma–Aldrich, Saint Quentin Fallavier, France) were each dissolved in sterile distilled water at a concentration of 0.545 M.

#### 2.1.1 Pharmacokinetics of OAS, NAS, and Serine in Lean Mice

In an initial pharmacokinetic study, mice received a single oral gavage of OAS at 1 g/kg body weight (BW) to evaluate its plasma kinetics, metabolism, and tissue distribution. Mice were euthanized at 15 min, 2 h, and 6 h post‐OAS administration (*n* = 5–14 per time point). Plasma, liver, and pancreas samples were collected and stored at −80°C for later quantification of OAS and its main metabolites, NAS and serine. To assess the intestinal absorption of NAS and serine, mice received a single oral gavage of serine or NAS at 0.6 g/kg (BW) and for each mouse submandibular blood was collected before, 15 min and 2 h after the oral bolus. Plasma was stored at −80°C for later quantification.

#### 2.1.2 Dose–Response and Time‐Course of OAS in Lean Mice

Mice were treated via oral gavage with 0.06, 0.2, 0.6, or 1 g/kg BW of OAS for five consecutive days, and control mice received H_2_O (*n* = 9 per dose group). On Day 5, OAS was co‐administered with glucose at the start of the oral glucose tolerance test (OGTT). To validate the acute response to OAS, mice were treated via oral gavage with 0.6 g/kg BW of OAS for five consecutive days and OAS was co‐administered with glucose at the start of the OGTT on Day 5 and compared to mice receiving only a single bolus of OAS at 0.6 g/kg BW administered 15 min before the OGTT. Control mice received H_2_O (*n* = 9 per group).

#### 2.1.3 Effects of NAS and Serine in Lean Mice

Mice received a daily oral gavage of NAS or L‐serine at 0.6 g/kg BW for five consecutive days, and control mice received H_2_O (*n* = 8–12 per group). On Day 5, NAS or L‐serine was co‐administered with glucose at the start of the OGTT. To ensure that systemic bioavailability of serine and NAS was not a limiting factor, mice received intraperitoneal (IP) injections of OAS, NAS, or L‐serine at 0.6 g/kg BW for five consecutive days, while control mice received sterile water (*n* = 8–9 per group). On Day 5, immediately prior to the OGTT, mice were administered a final IP injection of OAS, NAS, or L‐serine.

#### 2.1.4 Route of Administration Experiments

To assess the impact of the OAS administration route, OAS was delivered intraperitoneally or orally at 0.6 g/kg BW for 5 days, and control mice received H_2_O either orally or intraperitoneally (*n* = 8–10). On Day 5, OAS was either orally co‐administered with glucose or intraperitoneally a few seconds before glucose gavage at the start of the OGTT. To assess the impact of the glucose administration route, OAS was delivered orally at 0.6 g/kg BW for 5 days, and control mice received H_2_O (*n* = 9–10). On Day 5, OAS was administered orally a few seconds before glucose IP injection at the start of the intraperitoneal glucose tolerance test (IPGTT).

#### 2.1.5 Glucose‐Dependence Experiment

To assess whether the metabolic effect of OAS depends on the presence of glucose, mice received a daily oral gavage of OAS (0.6 g/kg body weight) or H_2_O (CTRL H_2_O) for five consecutive days (*n* = 20 per group). On Day 5, all mice were fasted for 6 h, after which submandibular blood was collected to measure basal insulin and glucose levels. Immediately afterward, the two groups were subdivided into four subgroups: two subgroups (*n* = 10 per group) received an oral 2 g/kg glucose load for OGTT (group “with glucose challenge”), while the two others (*n* = 10 per group) received H_2_O (group “without glucose challenge”). Submandibular blood was collected at T5 to measure GLP‐1 and GIP, and at T15 to measure insulin and glucose. All blood samples were collected into tubes containing EDTA (0.5 M) and a DPP‐IV inhibitor (4 mM; Merck, France). Plasma was separated by centrifugation (3000 × *g*, 15 min, 4°C) and stored at −80°C for subsequent insulin and gut peptide analysis.

#### 2.1.6 Administration of Exendin (9–39)

The GLP‐1 receptor antagonist exendin (9–39) was used to investigate the possible involvement of GLP‐1 in the glucose‐lowering effects of OAS. Mice received oral OAS at 0.6 g/kg BW for 5 days, and control mice received H_2_O. On Day 5, a subset of the H_2_O group and a subset of the OAS group received exendin (9–39) at 2 mg/kg BW via IP injection 30 min before OGTT. The remaining animals of each group received an equivalent volume of vehicle (*n* = 4–9 per group). OAS was then co‐administered with glucose at the start of OGTT. Submandibular blood samples from mice of OAS and OAS + exendin (9–39) groups were collected into tubes containing EDTA (0.5 M) at T0 and T15 of the OGTT. Plasma (3000 × *g*, 15 min, 4°C) was then stored at −80°C for subsequent insulin analysis.

#### 2.1.7 Effect of OAS on Glycaemic Regulation in a Diet‐Induced Prediabetic Mouse Model

A prediabetic model was established by feeding mice a high‐fat diet (HFD) (U8954 251‐HF, Safe, France) ad libitum for 4 weeks. A control group was fed a normal chow (NC). After 2 weeks, half of the HFD‐fed mice received a daily oral gavage of OAS at 0.6 g/kg BW (HFD + OAS), while the remaining groups received water (NC + H_2_O and HFD + H_2_O) (*n* = 9–10 per group). Two OGTTs were performed: the first after 2 weeks of diet (HFD or NC), and the second after two additional weeks of HFD and 2 weeks of treatment with OAS or H_2_O. On the day of the second OGTT, mice received OAS or H_2_O with glucose at the start of the OGTT. At euthanasia, mice were fasted for 2 h, and peripheral blood was collected 15 min after the final OAS or H_2_O administration into tubes containing EDTA (0.5 M) and a DPP‐IV inhibitor (4 mM; Merck, France). Plasma was separated by centrifugation (3000 × *g*, 15 min, 4°C) and stored at −80°C for subsequent insulin and GLP‐1 analysis.

### 2.2 Oral and Intraperitoneal Glucose Tolerance Test (OGTT and IPGTT), Insulin Tolerance Test (ITT), and Fasting‐Refeeding Test

After 6 h fasting, animals received an oral gavage (OGTT) or intraperitoneal injection (IPGTT) of glucose (2 g/kg BW for OGTT or 1 g/kg BW for IPGTT) and blood glucose levels were assessed at baseline (T0), and 15, 30, 60, 90 and 120 min post‐glucose administration. For experiments with normoglycaemic lean mice, OGTT and IPGTT were conducted on the 5th day of treatment. To determine whether GLP‐1 and insulin secretions were glucose‐dependent, blood samples were collected during the OGTT from the submandibular vein of normoglycaemic lean mice before glucose administration (2 g/kg BW), and at 5 and 15 min post‐glucose loading, respectively. For the fasting‐refeeding test, to prevent food neophobia mice (*n* = 6) received some customized high‐fat high‐sugar (HFHS) diet for 10 min during 4 days before the experiment. The HFHS diet contained HFD pellet (D12451 diet, Research Diets Inc., Denmark) and condensed milk in a 2:1 ratio. Each mouse served as its own control and underwent both experimental conditions. On the first test day of the experiment, after a 6‐h fast, mice received a single oral administration of H_2_O and, on a separate test day, oral administration of OAS (0.6 g/kg). Each administration was immediately followed by access to the HFHS diet for 10 min. Blood glucose levels were measured at T0 and at 10, 15, 30, and 60 min.

An insulin tolerance test (ITT) was conducted on the prediabetic mice on week 4. Mice were fasted for 4 h, received an IP injection of insulin (0.5 IU/kg BW), and the glucose levels were determined before (T0), and at 15, 30, 60, and 90 min after insulin administration.

### 2.3 Ins‐1 Cell Culture and Insulin Secretion Measurements In Vitro

Beta pancreatic INS‐1 cells were cultured in complete RPMI 1640 medium with 11 mM glucose, 1 mM sodium pyruvate, 50 μM 2‐mercaptoethanol, 2 mM glutamine, 10 mM HEPES, 100 IU/mL penicillin, 100 μg/mL streptomycin, and 10% heat‐inactivated fetal bovine serum (FBS). For the insulin secretion assay, INS‐1 cells were plated in 96‐well poly‐ornithine coated plates (Corning, France) and cultured under standard conditions (11 mM glucose, 10% FBS). On the assay day, cells were incubated with OAS at different concentrations (0, 0.125, 0.75, 1, 2 and 3 mM) for 30 min in Krebs Ringer Buffer (KRB)‐Hepes 0.1% BSA with 5.6 mM glucose. After incubation, supernatants were collected and frozen for insulin measurement. Cells were washed with D‐PBS, lysed with 0.1 N NaOH, and shaken until complete lysis. Protein quantification was performed using a colorimetric LOWRY method, with BSA as the calibration standard to normalize insulin measurements. Each condition was tested in sextuplicate. GLP‐1 (5, 10 nM) and the Forskolin/IBMX (FI) mix (0.1 μM/10 μM) served as positive controls to ensure cell responsiveness in 5.6 mM glucose.

### 2.4 Rat Islets Isolation, Culture, and Insulin Secretion Stimulation

Six‐week‐old male Wistar rats (Janvier Labs, Le Genest‐Saint‐Isle, France) were used for pancreatic islet isolation and housed at Metabrain Research (Les Ulis, France) following European guidelines (2010/63/UE—ETS 123) and CNREEA‐approved projects (APAFIS #0709, #2796, #4027). Rats were housed in groups of 4 per cage with a 12‐h daylight cycle and fed a standard diet (Safe, France) ad libitum. Pancreatic islets were isolated using the collagenase dissociation method, as previously described [44]. After isolation, islets were incubated under basal conditions with 2.8 mM glucose, or under stimulated conditions with 8 or 16.7 mM glucose, both in the presence or absence of OAS at 1.25 mM in 1% DMSO. At the end of incubation, supernatant samples were collected and stored at −80°C until insulin measurement. GLP‐1 (0.1 μM) was used as a positive control to ensure cell responsiveness at the different glucose concentrations (2.8, 8 and 16.7 mM).

### 2.5 GLP‐1, Glucose‐Dependent Insulinotropic Polypeptide (GIP), and Insulin Levels Measurement

Total GLP‐1, insulin and GIP concentrations in mouse plasma samples were measured using Merck ELISA kits (EMD Millipore Corporation France, that is, EZGLP1T‐36K, EZMRI‐13K and EZRMGIP‐55K, respectively), following the manufacturer's protocol. For GLP‐1, 20 μL of plasma were used, whereas 10 μL were used for insulin, and GIP. The insulinogenic index was calculated as the ratio of the change in insulin to the change in glucose between T0 and T15 (ΔInsulin_0–15_/ΔGlucose_0–15_).

In INS‐1 cell and pancreatic islet supernatant samples, insulin concentration was quantified by ELISA (Insulin rat high range ELISA Alpco Cat no. 80‐INSRTH‐E10) according to the manufacturer's recommendations, using 5 μL of supernatant.

### 2.6 OAS, NAS, and Serine Analysis by LC–MS/MS System

OAS, NAS, and serine were measured using liquid chromatography–tandem mass spectrometry (LC–MS/MS). Fifty microliters of internal standard solution (serine‐d3, 750 ng/mL in methanol) and 500 μL of acetonitrile with 1% formic acid (−20°C) were added to each tube containing ~50 mg of tissue or 50 μL of plasma. Tissue samples were homogenized with a ball mill TissueLyser LT (Qiagen, Courtaboeuf, France) using a 5 mm stainless steel bead for 20 min at 50 Hz, while plasma samples were vortexed. Tubes were centrifuged (10 000 rpm, 10 min, 4°C). One hundred microliters of supernatant were desalted using ZipTip C18 pipette tips (Merck, Darmstadt, Germany) before injection. Chromatography was performed with an UltiMate 3000 Quaternary Rapid Separation Pump LPG‐3400RS (Thermo Scientific Dionex, Les Ulis, France) using an ACQUITY UPLC BEH HILIC column (2.1 × 100 mm, 1.7 μm; Waters, Guyancourt, France) at 40°C. A 10 μL injection was eluted in gradient mode with water (pH 2.1, formic acid) and acetonitrile: 95% acetonitrile for 1 min, decreasing to 77% at 8 min, followed by a 3 min re‐equilibration. Flow rate was 0.4 mL/min during 11 min total. Compounds were detected with a triple quadrupole Quantiva mass spectrometer (Thermofisher, France) equipped with a heated electrospray ionization source. Nitrogen (N2‐45 nitrogen generator, VWR International, Fontenay‐sous‐Bois, France) was used as sheath and auxiliary gas. The HESI source operated in positive ionization mode with the following parameters: sheath gas: 20; aux. gas: 5; sweep gas: 1; spray voltage: 3.3 kV; ion transfer tube temp.: 325°C; vaporizer temp.: 100°C. Data acquisition was in multiple reaction monitoring mode with monitored ions (m/z) for each compound: OAS: 148.3 → 88.1 (11) and 148.3 → 106.1 (11); NAS: 148.4 → 60.0 (17) and 148.4 → 106.1 (10); serine: 106.1 → 59.9 (11) and 106.1 → 42.1 (22); serine‐d3 (IS): 109.3 → 63.1 (12) and 109.3 → 45.1 (22). Retention times were 1.0, 6.0, and 6.3 min for NAS, OAS, and serine/serine‐d3, respectively. Data acquisition and processing were performed using Chromeleon v6.80 (Thermo Scientific Dionex, Les Ulis, France) and Xcalibur v3.0.63 (Thermofisher, France). A calibration curve with one zero and eight calibration standards was included in each experiment series. Quantitation was based on peak area ratios relative to the internal standard using mean least squares linear regression with a 1/× weighting factor. The lower limit of quantification (LLOQ) was 10 ng/mL for NAS and OAS, and 50 ng/mL for serine. The upper limit of quantification (ULOQ) was 1500 ng/mL for all three compounds.

### 2.7 Statistics Analysis

Statistical analysis was performed using GraphPad Prism (v7; San Diego, CA). OGTT curves are presented as mean ± 95% confidence interval and other data are presented as mean ± SEM. Normal distribution was verified via the D'Agostino & Pearson test. One‐way ANOVA or Kruskal–Wallis was used for experiments with more than two groups, while unpaired *T*‐test, Mann–Whitney test, or Wilcoxon paired‐test were applied for two‐group comparisons. Specific tests are detailed in figure legends.

### 2.8 Data and Resource Availability

The data sets generated during the current study are available upon request. The resources are available upon request.

## 3 Results

### 3.1 OAS Specifically Improves Glucose Tolerance in Lean Mice in a Dose Depend Manner

**FIGURE 1:**
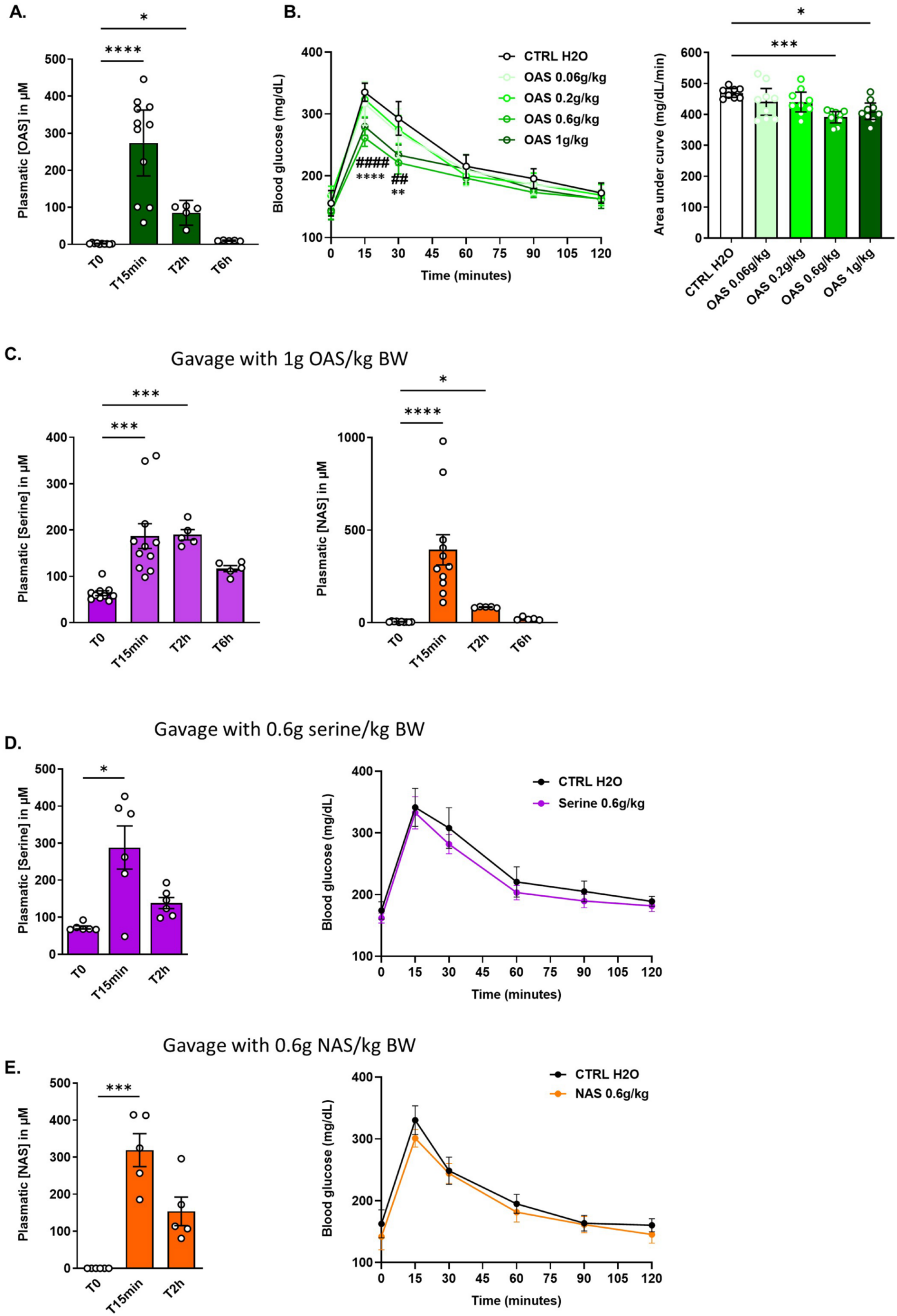
Effect of OAS on postprandial glycaemia and its pharmacokinetics in a healthy context. (A) Plasma levels of OAS at baseline (T0), 15 min, 2 h, and 6 h following a single oral dose of OAS (1 g/kg of body weight [BW]) (*n* = 5–11). (B) Dose–response of OAS on blood glucose levels during an OGTT after 5 days of daily gavage with H_2_O (CTRL H_2_O) or OAS at different concentrations (0.06, 0.2, 0.6, and 1 g/kg BW) (*n* = 9). OGTT curves (left panel) and area under the curve (AUC) (right panel). (C) Plasma levels of serine (left panel) or NAS (right panel) at T0, 15 min, 2 h, and 6 h following a single oral dose of OAS (1 g/kg BW) (*n* = 5–12). (D) Plasma levels of serine at T0, 15 min, 2 h, following a 5 day of daily gavage with serine (0.6 g/kg BW) (*n* = 5–6) (left panel). Blood glucose levels during an OGTT following 5 days of daily gavage with H_2_O or serine (0.6 g/kg BW) (*n* = 10–12) (right panel). (E) Plasma levels of NAS at T0, 15 min, 2 h, following a 5 day of daily gavage with NAS (0.6 g/kg BW) (*n* = 5–6) (left panel). Blood glucose levels during an OGTT following 5 days of daily gavage with H_2_O or NAS (0.6 g/kg BW) (*n* = 8–9) (right panel). OGTT curves are presented as mean ± 95% confidence interval and all the other data are presented as mean ± SEM. Significance was determined using two‐way ANOVA (OGTT curves), one‐way ANOVA (OGTT AUC), and Kruskal–Wallis test for all plasma concentrations with the following *p*‐values: *p < 0.05, **p < 0.01, ***p < 0.005, ****p < 0.0001. In panel B, * indicates comparisons between OAS 0.6 g/kg and CTRL H_2_O; # indicates comparisons between OAS 1 g/kg and CTRL H_2_O with **p < 0.01, ****p < 0.0001, ^##^ *p* < 0.01 and ^####^ *p* < 0.0001. BW, body weight; NAS, *N*‐acetyl‐serine; OAS, *O*‐acetyl‐serine; OGTT, oral glucose tolerance test.

To evaluate the pharmacokinetics of OAS, we first measured its plasma concentration at multiple time points after administering a single oral dose of 1 g/kg to lean mice. The baseline plasma concentration was 2.3 ± 0.4 μM (Figure 1A). It increased markedly by approximately 100‐fold at 15 min (reaching 273.8 ± 39.9 μM) and by 35‐fold at 2 h post‐administration (reaching 85.3 ± 12.1 μM), before returning to baseline levels by 6 h (Figure 1A). To investigate the effect of OAS on postprandial glycaemia, mice were treated with increasing doses of OAS (0.06, 0.2, 0.6, and 1 g/kg/day) for 5 consecutive days, and glucose tolerance was assessed to identify the lowest effective dose. On Day 5, OAS was co‐administered with glucose at the start of the OGTT so that the expected glucose peak (around 15 min) would coincide with the observed peak in plasma OAS concentration following its oral administration. OAS at doses of 0.6 and 1 g/kg significantly reduced blood glucose levels at T15 and T30 following glucose administration compared to control mice (Figure 1B, left panel). This glucose‐lowering effect was dose‐dependent, as indicated by a significant reduction in the area under the curve (AUC) for both the 0.6 and 1 g/kg groups but not for the other groups (Figure 1B, right panel). A single dose of 0.6 g/kg OAS was sufficient to significantly reduce glycaemia, demonstrating the acute effect of OAS (Figure S1A). A similar glycaemia‐lowering effect of OAS was observed following a mixed meal in a fasting‐refeeding test (Figure S1B), emphasizing the impact of OAS on postprandial glycaemia. In vitro, OAS is pH‐sensitive and can be spontaneously converted into NAS or back to its precursor serine when pH values are above 7 [45]. Given that the metabolic fate of OAS in humans remains unknown, we examined whether serine or NAS could be detected in plasma following OAS administration. The baseline plasma serine concentration was 63.4 ± 5.2 μM (Figure 1C, left panel), and the NAS concentration was 4.8 ± 0.8 μM (Figure 1C, right panel). Following a single oral dose of OAS (1 g/kg), serine levels tripled significantly at 15 min, reaching 190 μM, and remained stable over the subsequent 2 h (Figure 1C, left panel). In contrast, NAS exhibited a marked and significant increase at 15 min (400 μM) but declined rapidly by 2 h, returning to baseline levels by 6 h (Figure 1C, right panel). Oral delivery of serine or NAS (0.6 g/kg) significantly increased circulating levels of both compounds, reaching 288 and 236 μM, respectively, indicating efficient intestinal absorption of both molecules (Figure 1D,E, left panels). To assess whether these metabolites contributed to the glucose‐lowering effect, mice were treated with either serine or NAS (0.6 g/kg/day) for 5 days. On Day 5, each compound was co‐administered with glucose at the start of the OGTT. Neither treatment significantly altered glucose levels during the OGTT compared to controls (Figure 1D,E right panels), suggesting that the glucose‐lowering effect is specific to OAS and not mediated by its known derivatives.

### 3.2 Glycaemic Regulation by OAS Is Independent of OAS and Glucose Delivery Routes

**FIGURE 2:**
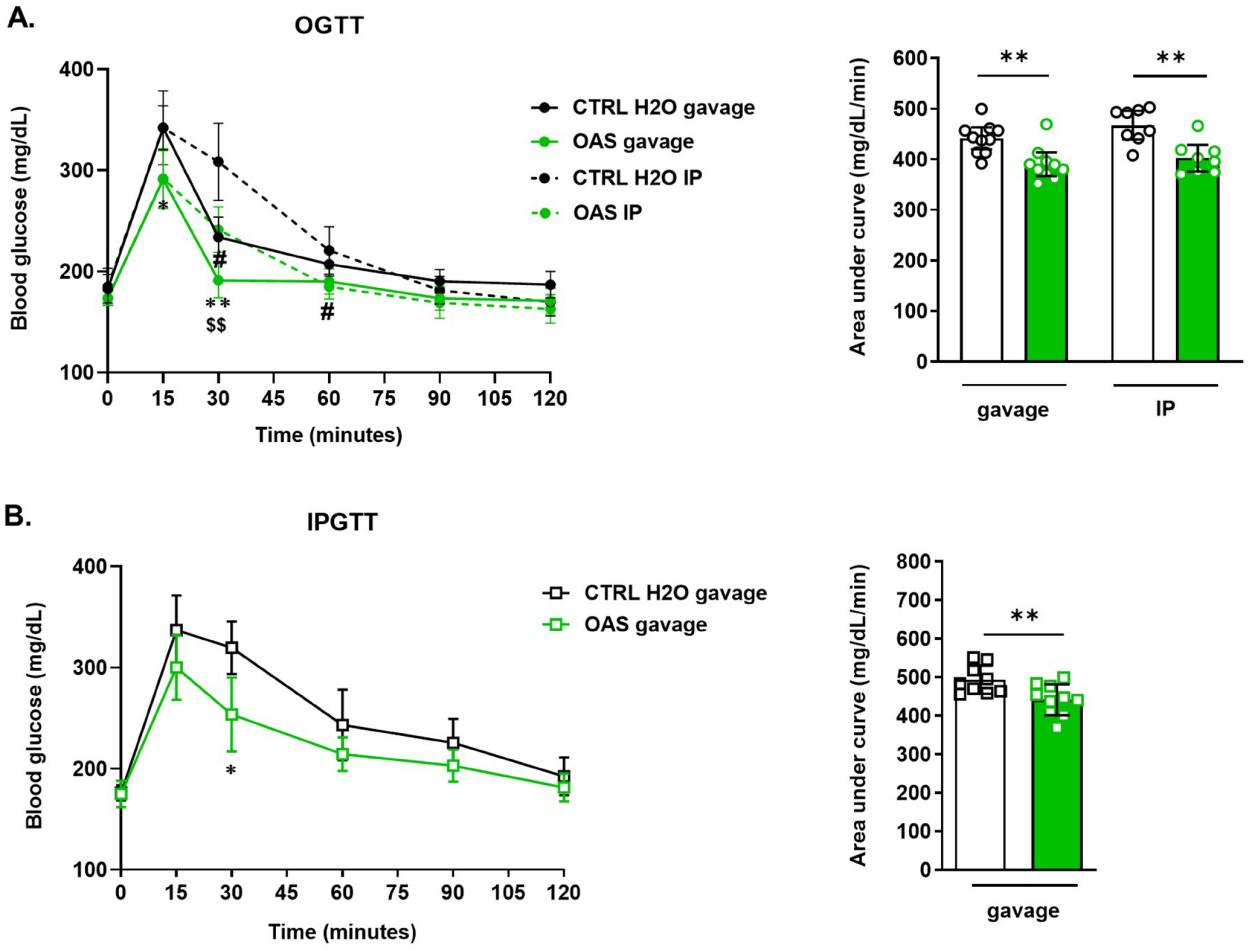
Glycaemic regulation by OAS is independent of both OAS and glucose delivery routes. (A) Blood glucose levels during OGTT after 5 days of daily gavage or IP administration with H_2_O or OAS at 0.6 g/kg BW, (*n* = 8–10). OGTT curves (left panel) and AUC (right panel). (B) Blood glucose levels during IPGTT after 5 days of daily gavage with H_2_O or OAS at 0.6 g/kg BW, (*n* = 9–10). IPGTT curves (left panel) and AUC of IPGTT (right panel). OGTT curves are presented as mean ± 95% confidence interval and AUC are presented as mean ± SEM. Significance was determined using two‐way ANOVA (OGTT curves) and *T*‐test (AUCs) with the following *p*‐values: *p < 0.05, **p < 0.01, ^#^ *p* < 0.05 and ^$$^ *p* < 0.01. In panel A, # indicates the comparison between OAS IP and CTRL‐H_2_O IP, * the comparison between OAS gavage and CTRL‐H_2_O gavage and $ the comparison between OAS gavage and OAS IP. IP, intraperitoneal; IPGTT, intraperitoneal glucose tolerance test; OAS, *O*‐acetyl‐serine; OGTT, oral glucose tolerance test.

During a glucose tolerance test, the route of both OAS and glucose delivery (oral versus IP) can significantly affect glycaemic regulation due to factors like intestinal glucose absorption and intestinal incretin‐mediated insulin secretion (such as GIP or GLP‐1). To determine whether intestinal detection and transport of OAS are required for post‐prandial glycaemia reduction, we compared the effects of 5 days of IP versus oral administration of OAS (0.6 g/kg) on plasma glucose levels (Figure 2A). During the OGTT, significantly lower glucose concentrations were measured at T15 and T30 in orally gavaged mice. In IP‐injected mice, glucose levels showed a strong trend toward reduction at T15 (*p* = 0.054) and were significantly lower at T30 and T60 compared to their respective CTRL‐H_2_O groups (Figure 2A, left panel). In addition, at T30, orally delivered OAS resulted in a significantly greater reduction in glycaemia than IP delivery. To confirm that the route of delivery does not alter the specificity of OAS relative to its metabolites, we also performed 5 days of IP administration of NAS and serine (0.6 g/kg). Under these conditions, only IP‐administered OAS significantly reduced glucose levels at T15 and T30 (Figure S2) confirming that neither NAS nor serine replicate the glucose‐lowering effects of OAS.

Both OAS‐treated groups showed a significant decrease in AUC_OGTT‐glucose_, regardless of delivery mode (Figure 2A, right panel), indicating that OAS's effect on postprandial glycaemia is independent of its administration route.

Next, to assess whether the glucose administration route influences OAS's effect on postprandial glycaemia, mice treated orally with OAS (0.6 g/kg) for 5 days underwent an IPGTT on Day 5 (Figure 2B). OAS‐treated mice showed significantly lower glycaemia at 30 min post‐glucose administration (Figure 2B, left panel) and a lower AUC_IPGTT‐glucose_ compared to CTRL‐H_2_O mice (Figure 2B, right panel). These findings suggest that OAS regulates glycaemia independently of the glucose administration mode.

### 3.3 OAS Enhances Glucose‐Induced Insulin Secretion in the Pancreas

**FIGURE 3:**
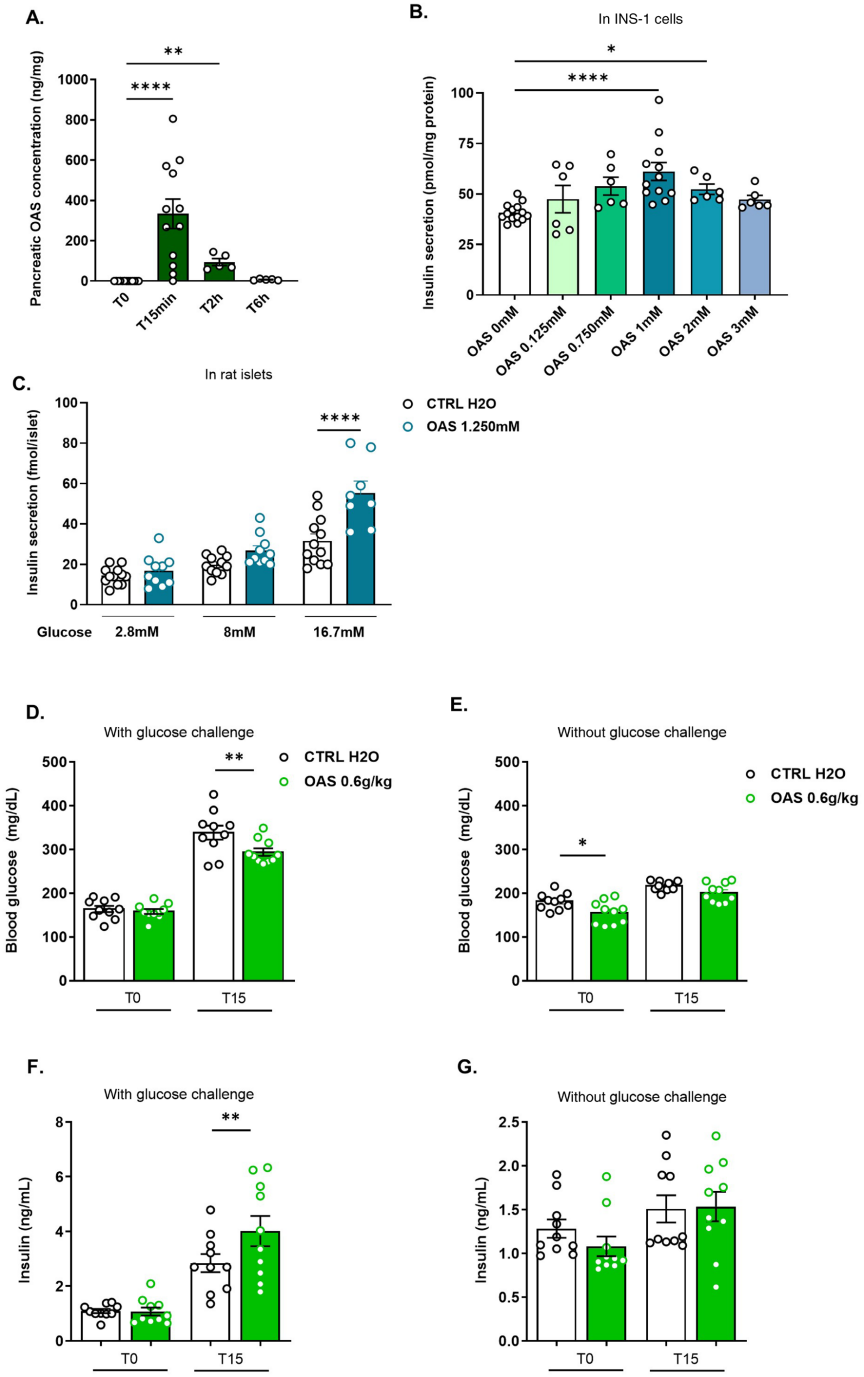
OAS enhances glucose‐induced insulin secretion in the pancreas. (A) Pancreatic levels of OAS at 0, 15 min, 2 and 6 h following a single gavage of OAS (1 g/kg BW) (*n* = 5–14). (B) Insulin secretion from INS‐1 cells after 30 min of incubation in the presence of glucose (5.6 mM), with or without OAS (0 to 3 mM), (*n* = 6–14 in two independent experiments). (C) Insulin secretion from Wistar rat islets after 30 min of OAS treatment (1.25 mM) in the presence of different concentrations of glucose (2.8, 8 and 16.7 mM). (*n* = 8–12). Blood glucose levels after 5 days of daily gavage with H_2_O or OAS at 0.6 g/kg BW at T = 0 (T0) and T = 15 min (T15) following a challenge (D) with or (E) without glucose (2 g/kg BW), (*n* = 10). Plasma insulin levels were measured in blood samples collected at T0 and T15 following the challenge (F) with or (G) without glucose (2 g/kg BW), (*n* = 10). Data are presented as mean ± SEM. Significance was determined using one‐way ANOVA (A, B, D–F), Kruskal–Wallis test (G) and *T*‐test between CTRL H_2_O and OAS (C), with the following *p*‐values: *p < 0.05, **p < 0.01, ***p < 0.005, ***p < 0.0001. OAS, *O*‐acetyl‐serine.

Insulin, a key glycaemia regulator, is secreted by pancreatic β‐cells in response to stimuli, including amino acids. In our study, intraperitoneal OAS delivery reduced postprandial glycaemia, suggesting a potential direct pancreatic action. To test this, we measured pancreatic OAS levels at different time points post oral administration of OAS and assessed its effect on insulin secretion in vitro, using INS‐1 cells and rat islets, and in vivo. Pancreatic OAS levels increased approximately 100‐fold from baseline following a single oral dose of 1 g/kg in lean mice, peaking at 334 ng/mg tissue at 15 min (Figure 3A). Levels then declined over 2 to 6 h, returning close to basal values (94 ± 10.6 ng/mg tissue and 7.9 ± 0.9 ng/mg tissue respectively). Notably, OAS increase was pancreas‐specific, with no significant rise in liver OAS levels (Figure S3). In INS‐1 cells cultured with 5.6 mM glucose, as expected GLP‐1 (5 nM) and Forsk‐IBMX mix (1 μM/10 μM) stimulated insulin secretion by 2.4‐ and 3.8‐fold, respectively (Figure S4A) and OAS (1–2 mM) increased insulin secretion by 1.5‐ and 1.3‐fold compared to untreated cells (Figure 3B). To investigate the interaction between OAS and glucose during postprandial glycaemia, we used rat pancreatic islets. As expected in islets [46], GLP‐1 (0.1 μM) enhanced insulin secretion at 8 and 16.7 mM glucose but not at 2.8 mM (Figure S4B). OAS had no significant effect on insulin secretion in presence of 2.8 or 8 mM glucose. However, at 16.7 mM glucose, OAS increased insulin secretion by 1.8‐fold (Figure 3C). These findings show that OAS reaches the pancreas and stimulates insulin secretion in a glucose‐dependent manner. Since OAS stimulates insulin secretion in a glucose‐dependent manner in vitro, we examined its interaction with glucose in vivo. Mice received oral OAS (0.6 g/kg of BW) or H_2_O (CTRL‐H_2_O) for 5 days. On Day 5, OAS was co‐administered either with glucose (regular OGTT) or with water (control condition to assess OAS effects in the absence of glucose). At T0, blood glucose levels showed no or slight significant differences between OAS‐treated and CTRL‐H_2_O mice in either experiment (Figure 3D,E). However, at T15, a significant 12% decrease in glycaemia was observed in mice challenged with glucose and treated with OAS, compared to CTRL‐H_2_O mice group (Figure 3D). Furthermore, at T15 post‐glucose administration, the reduction in glycaemia observed in OAS‐treated mice was associated with a marked 3.7‐fold elevation in plasma insulin levels significantly exceeding those of CTRL‐H_2_O mice (Figure 3F) leading to an increased insulinogenic index (insulinogenic index at T15: OAS vs. CTRL‐H_2_O, 12.5 vs. 6.1, respectively; *p* = 0.04) reflecting an amplified early‐phase insulin secretion. Without a glucose challenge (Figure 3G), insulin levels remained unchanged, confirming that glucose is necessary for OAS to affect glycaemia in vivo, as observed in vitro.

### 3.4 OAS Regulates Glycaemia Only Partly Through GLP‐1 Signaling

**FIGURE 4:**
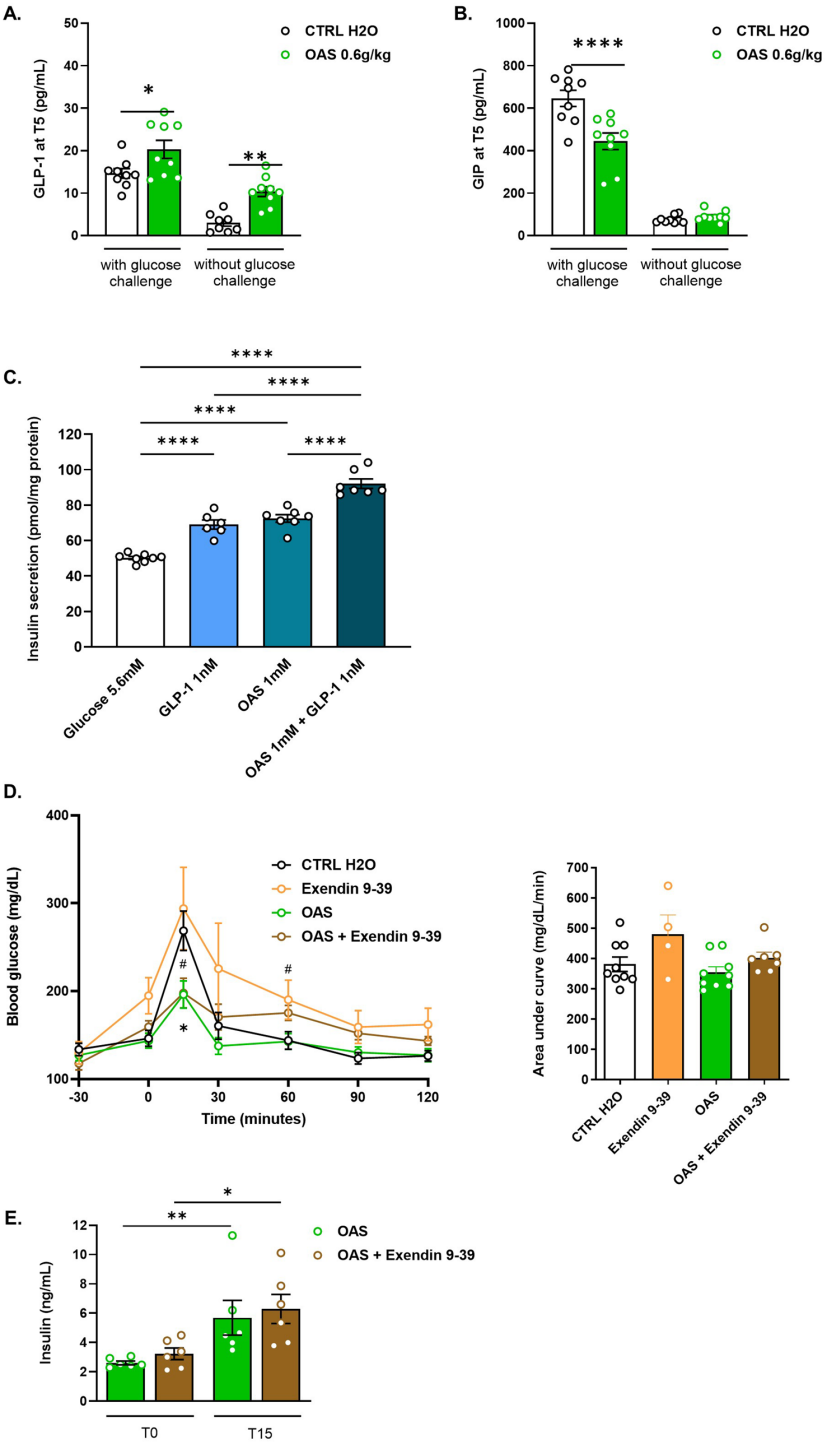
OAS modulates GLP‐1 but partly synergizes with it to stimulate insulin secretion. Plasma levels of (A) GLP‐1 and (B) GIP measured in blood samples collected at T = 5 min (T5) following a challenge with or without glucose. (C) Insulin secretion from INS‐1 cells after 30 min of incubation in the presence of glucose (5.6 mM), with GLP‐1 (1 mM) or OAS (1 mM) or OAS (1 mM) + GLP‐1 (1 mM), (*n* = 6–8). (D) Blood glucose levels during OGTT after 5 days of daily gavage with OAS at 0.6 g/kg BW with or without exendin (9–39) being injected 30 min before an oral glucose tolerance test in lean mice. OGTT curves. (*n* = 4–9 per group). The left panel shows OGTT curves; the right panel shows the area under the curve (AUC). (E) Insulin levels at T0 and T15 of the OGTT after 5 days of daily gavage with OAS (0.6 g/kg BW) with or without exendin (9–39) injection (*n* = 6). OGTT curves are presented as mean ± 95% confidence interval and other data are presented as mean ± SEM. Significance was determined using a *T*‐test between CTRL H_2_O and OAS (A, B) and a one‐way ANOVA (C) with the following *p*‐values: *p < 0.05, **p < 0.01, ***p < 0.005, ***p < 0.0001. For (D) a Kruskal–Wallis test was performed at each time‐point for non‐normally distributed OGTT data, * indicating comparisons between OAS and CTRL H_2_O and # indicating comparisons between OAS + exendin 9–39 and CTRL H_2_O, with *; ^#^ *p* < 0.05. For (E) Mann–Whitney tests were performed between T0 and T15 with *p < 0.05 and with **p < 0.01. GIP, Glucose‐dependent insulinotropic polypeptide; GLP‐1, Glucagon‐like peptide‐1; OAS, *O*‐acetyl‐serine.

To clarify the role of intestinal incretins, blood was sampled at T5 of the OGTT of the previous experiment to measure GLP‐1 and GIP. As expected, both GLP‐1 and GIP levels increased at T5 in response to glucose challenge (Figure 4A,B). Interestingly, GLP‐1 levels were significantly higher in OAS‐treated mice than in non‐treated CTRL mice, both with and without glucose challenge (Figure 4A), while GIP levels did not increase in response to OAS treatment and even decreased significantly at T5 in the context of a glucose challenge (Figure 4B). Since OAS directly stimulates insulin secretion, similar to GLP‐1, we tested whether their effects are additive using INS‐1 cells. Combining 1 mM OAS with 1 nM GLP‐1 significantly increased insulin secretion by 1.3‐fold compared with OAS or GLP‐1 alone, and by 1.8‐fold compared with glucose alone (Figure 4C), suggesting that OAS and GLP‐1 activate insulin secretion through distinct pathways and that OAS and GLP‐1 have an additive effect on insulin secretion in vitro. We next tested whether the glucose lowering effect of OAS depends at least partly on GLP‐1 signaling. Mice, treated or not with OAS, received the GLP‐1 receptor antagonist exendin (9–39) 30 min before OGTT. Consistent with previous reports [47], exendin (9–39) increased the glycaemic excursion compared to non‐treated CTRL H_2_O mice (Figure 4D). Importantly, OAS still improved oral glucose tolerance at T15 in mice pretreated with exendin (9–39), although the effect was slightly reduced after the first 30 min following OAS/glucose administration compared to mice treated with OAS alone (Figure 4D). In addition, increase in circulating levels of insulin was similar at T15 between OAS and OAS + exendin (9–39) treated mice (Figure 4E). Together, these data indicate that exendin (9–39) only partially attenuated the glucose‐lowering effect of OAS, while the early insulin response remained unchanged, suggesting that GLP‐1 receptor signaling is not the primary mediator of OAS‐induced improvements in glycaemic control, particularly during the early postprandial phase.

### 3.5 OAS Treatment Restores Glucose Tolerance in Prediabetes Context

**FIGURE 5:**
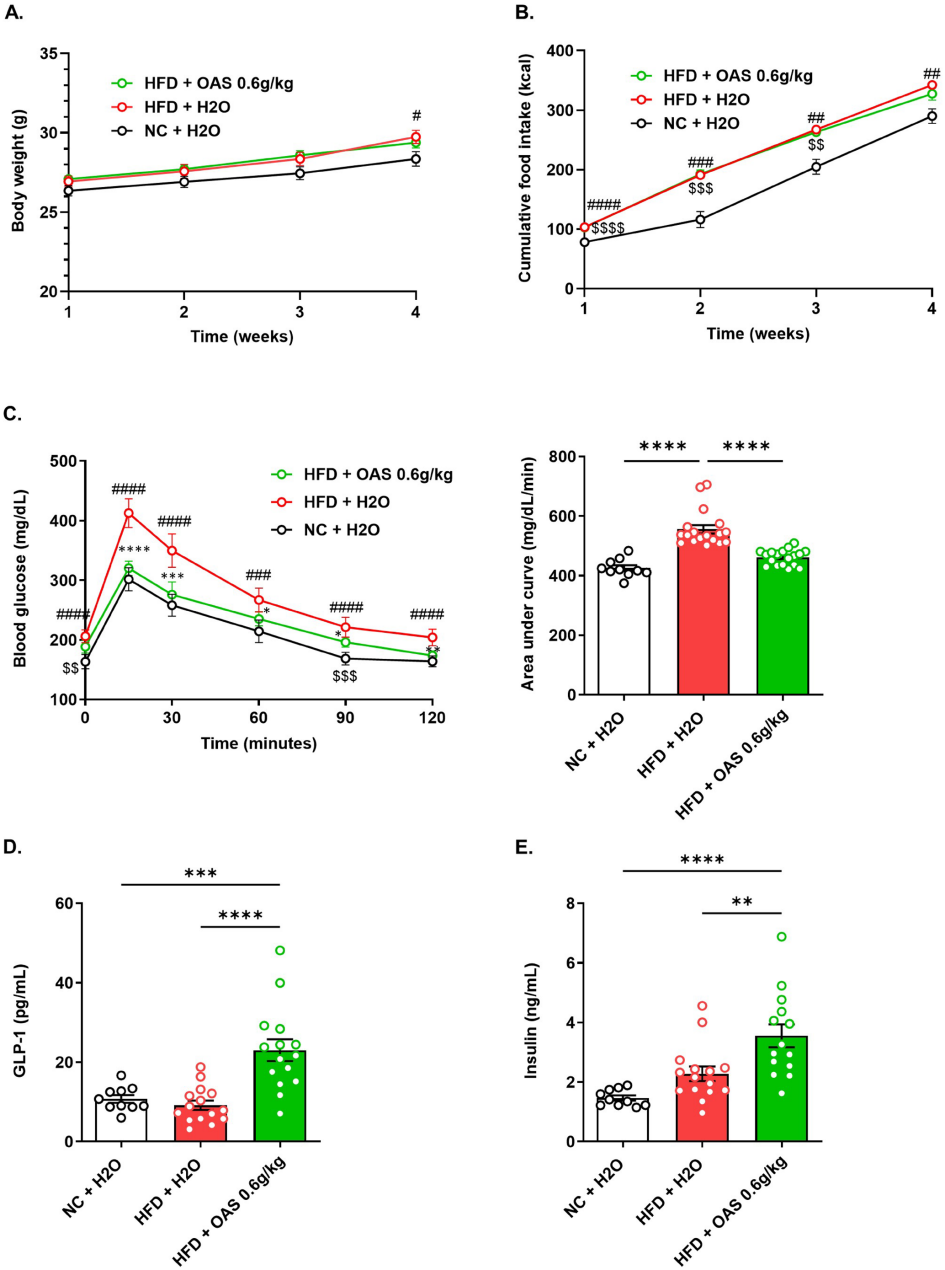
OAS treatment restores glucose tolerance in a prediabetic context. (A) Body weight and (B) cumulative food intake over a 4‐week period in mice fed either a normal chow (NC) or high‐fat diet (HFD). After 2 weeks, half of the HFD‐fed mice received a daily oral gavage of *O*‐acetyl‐serine (OAS) at 0.6 g/kg body weight (HFD + OAS), while the remaining groups received water (NC + H_2_O and HFD + H_2_O). (C) Blood glucose levels during an oral glucose tolerance test (OGTT) performed after 4 weeks of diet, with the last 2 weeks including daily gavage of either H_2_O or OAS (*n* = 10–18). The left panel shows OGTT curves; the right panel shows the area under the curve (AUC). (D) Plasma GLP‐1 and (E) insulin levels measured 15 min after the final administration of H_2_O or OAS (*n* = 10–15 for GLP‐1, *n* = 8–13 for insulin). OGTT data are presented as mean ± confidence interval, while all other data are shown as mean ± SEM. Statistical significance was assessed using two‐way ANOVA for body weight, food intake, and OGTT curves; Kruskal–Wallis test for OGTT AUC and GLP‐1 levels; and one‐way ANOVA for insulin levels. * indicates comparison between HFD + H_2_O and HFD + OAS; # indicates comparison between HFD + H_2_O and NC + H_2_O; $ indicates comparison between HFD + OAS and NC + H_2_O in the body weight, cumulative food intake and OGTT curves. *p < 0.05, **^,$$,##^ *p* < 0.01, ***^,$$$,###^ *p* < 0.001, ****^,^^*$$$$*^^, ####^ *p* < 0.0001.

The curative effect of OAS was evaluated in a mouse model of prediabetes induced by HFD. Animals were fed either a NC or HFD for 4 weeks. NC‐fed mice and a subset of HFD‐fed mice were treated with water, while the remaining HFD‐fed mice received a daily oral delivery of OAS at 0.6 g/kg BW (HFD + OAS 0.6 g/kg) during the last 2 weeks of HFD feeding. OAS administration did not affect body weight gain and HFD‐induced increase in food intake (Figure 5A,B). Glucose intolerance onset was assessed after 2 weeks of HFD feeding before the beginning of OAS treatment. At this time point, HFD‐fed mice already exhibited significantly higher glucose intolerance compared to NC‐fed mice (Figure S5). After 4 weeks of HFD exposure, HFD‐fed mice showed higher glucose level intolerance than NC‐fed mice, as evidenced by elevated glucose levels (Figure 5C, left panel). Interestingly, mice treated with OAS displayed a significant reduction in circulating glucose levels at all time points of the OGTT (Figure 5C, left panel), along with a 16% decrease in the AUC_OGTT‐glucose_ (Figure 5C, right panel). However, circulating glucose levels in response to the insulin tolerance test remained unchanged among the three groups (Figure S6). These findings indicate that OAS treatment improves glucose intolerance and restores normal glycaemia, independently of any change in insulin sensitivity. Consistent with previous results obtained in a non‐diabetic mouse model treated for 5 days with OAS, the reduction in glycaemia observed here was associated with increases in plasma levels of GLP‐1 (a 2.5‐fold increase) (Figure 5D) and insulin (a 1.7‐fold increase) (Figure 5E) compared to HFD + H_2_O mice.

## 4 Discussion

In the current study, we demonstrated that OAS improves post‐prandial glycaemia by enhancing insulin secretion in both healthy and prediabetic mouse models. Our data indicate that OAS increases GLP‐1 secretion in vivo, and although GLP‐1 signaling contributes to its action, it does not fully account for OAS's effect on glycaemic control. Instead, OAS also acts directly on pancreatic β‐cells, stimulating insulin secretion in a glucose‐dependent manner. Together, these findings suggest that OAS is a glucose‐dependent insulin secretagogue that improves glucose homeostasis in both lean and prediabetic mice.

In humans, OAS is found in biological fluids (e.g., urine, plasma), and its presence is attributed to both dietary intake of plants, vegetables and metabolic activity of the gut microbiota. As a key conserved intermediate in the biosynthesis of cysteine within plant and bacterial metabolic networks, OAS plays a central role in cysteine synthesis and sulfur nutrition [48, 49, 50, 51]. Increasing evidence suggest that bacterial and gut microbiota‐derived metabolites, including amino acid‐related compounds, contribute to glucose regulation and TD2, primarily by stimulating GLP‐1 and insulin secretion among others mechanisms [52]. This underscores the importance of bacterial and food‐derived metabolites, such as OAS, in the glycaemia regulation, and particularly in the processes of GLP‐1 and insulin secretion. In the present study, we demonstrated that the administration of OAS, a neutral derivative of serine, improved glucose tolerance in mice. Previous findings highlighted that fact that altered serine metabolism has been observed in insulin‐resistant mouse models and diabetic patients suggesting a potential preventive role for serine in glycaemic dysregulation [18, 23, 24, 25, 26, 27]. However, few studies have explored serine supplementation's effects on hyperglycaemia in mice, with mixed results despite high doses or extended treatments [27, 30, 53]. For example, 280 mg/day for 40 weeks reduced AUC_OGTT_ by 40% in a T1D model [30], whereas 40 mg/day for 12 weeks led to only a 13% reduction in HFD‐fed mice [53]. A 10‐fold increase in dietary serine (120 mg/day) even slightly elevated fasting glucose in db/db mice after 14 weeks [27]. In contrast, our study achieved a 15%–20% reduction in postprandial glycaemia in HFD‐fed mice with just 15 mg/day for 2 weeks, or as little as 1 day in healthy mice. These findings highlight OAS's superior efficacy and rapid action over serine, and suggest OAS's effects are unlikely due to its conversion into serine. Furthermore, although OAS administration caused transient increases in circulating serine, 5‐day serine treatments did not reproduce OAS's glycaemic benefits, indicating that these effects cannot be attributed to serine. Similarly, we found that OAS can be converted into NAS in vivo. However, NAS supplementation also failed to mimic OAS's impact on glycaemic control.

While investigating how OAS regulates postprandial glycaemia, we found that OAS stimulates GLP‐1 secretion independently of glucose. GLP‐1 is produced by enteroendocrine L‐cells in the gastrointestinal tract and can be triggered by various secretagogues, including glucose and specific amino acids [54, 55]. Similar to our findings with OAS, a previous study showed that amino acids like glutamine, alanine, and serine can induce GLP‐1 secretion in vitro more effectively than glucose [56]. However, the exact mechanisms by which ingested amino acids trigger GLP‐1 release are not fully characterized, and it remains unclear whether they stimulate secretion by activating luminal or basolateral sensors. Recent studies have suggested that amino acids are rapidly absorbed and primarily stimulate GLP‐1 secretion from the vascular side [57]. In our study, we clearly demonstrate that OAS is efficiently absorbed, reaching maximum plasma levels within 15 min of oral administration. Thus, OAS may stimulate GLP‐1 secretion through both apical and basolateral pathways. The effect of OAS following both intraperitoneal and oral administration supports this possibility of a basolateral mechanism of action.

Numerous transporters, mostly electrogenic, facilitate the intestinal transport of related amino acids and are expressed in enterocytes and enteroendocrine L‐cells along the gastrointestinal tract [58, 59]. OAS, a neutral amino acid derivative of serine, is likely transported by SNAT2 (sodium neutral amino acid transporter 2, SLC38A2) and B0AT1 (broad neutral amino acid transporter 1, SLC6A19), two transporters abundantly expressed in enteroendocrine cells and responsible for neutral amino acid uptake [56]. However, as SLC6A19‐deficient mice exhibit increased GLP‐1 secretion [57], B0AT1 may not be a key mediator of GLP‐1 release in response to OAS, warranting further studies to elucidate its mechanism of action on enteroendocrine cells.

Importantly, we observed in our study a modest attenuation of OAS's glucose‐lowering effect by exendin (9–39) suggesting that GLP‐1 receptor signaling contributes to its action but does not fully account for the observed improvement in glycaemic control. This is consistent with our in vitro findings in INS‐1 cells, where OAS and GLP‐1 together exerted a greater effect on insulin secretion than either alone, suggesting a possible additive or synergistic interaction. But more importantly, this experiment also demonstrated that OAS can directly enhance insulin secretion in pancreatic β‐cells, independently of GLP‐1. This aligns with the observation that plasma glucose reduction was similar after both oral and intraperitoneal OAS administration, indicating that gut‐derived factors are not the sole regulators of glycaemia in response to OAS, although, as previously discussed, intraperitoneal administration might still activate basolateral GLP‐1. Additional evidence in our study comes from rat pancreatic islets, where OAS stimulated insulin secretion in the presence of glucose. This glucose‐dependent insulin response to OAS is consistent with our in vivo findings. In this respect, OAS appears to act similarly to other amino acids, such as arginine and leucine, which are known to potentiate glucose‐stimulated insulin secretion when glucose levels are sufficient [13, 60, 61, 62]. Amino acids can stimulate insulin secretion through two distinct pathways. The triggering pathway (K^+^‐ATP channel‐dependent) involves plasma membrane depolarization, leading to an influx of Ca^2+^ and subsequent insulin granules exocytosis. In contrast, the amplifying pathway (K^+^‐ATP channel‐independent) potentiates the insulin secretion response to initial signals, such as glucose‐induced calcium signaling [61, 63]. This amplification may occur through mechanisms such as amino acid co‐transport with sodium or the uptake of cationic amino acids. Our findings suggest that OAS primarily influences insulin secretion through the amplifying pathway, as its effect is glucose‐dependent, as shown by both the in vitro and in vivo experiments. Additionally, our study revealed that OAS is metabolized into NAS and serine, suggesting that this metabolic process may support the Krebs cycle by providing serine. Consequently, OAS metabolism could contribute to an increase in the ATP/ADP ratio in β‐cells, leading to the closure of K^+^‐ATP channels.

To date, OAS has rarely been quantified in the plasma of mammals. To our knowledge, our study is among the first to establish a specific method for its detection not only in plasma but also in various tissues. The physiological concentration of OAS in humans remains poorly characterized, with only a few reports documenting its presence in blood, and none in tissues [38, 39]. Moreover, while a recent study reported OAS among several metabolites altered in the plasma of tuberculosis patients [64], no study has addressed its variation under broader pathological conditions such as diabetes or prediabetes. This knowledge gap is significant because OAS lies at the interface of host and microbial metabolism and commensal bacteria carrying the serine acetyltransferase (*cysE*) gene are able to convert serine into OAS [65]. Given the increasing recognition of gut microbiota dysbiosis in the pathogenesis of type 2 diabetes, it would be particularly interesting to determine whether the abundance of OAS‐producing bacteria differs between diabetic, prediabetic, and normoglycaemic individuals. Such analyses could provide insights into whether circulating OAS reflects microbial activity and whether altered OAS metabolism contributes to the protective or pathogenic effects of the microbiota on glucose homeostasis. Moreover, OAS has also been detected in vegetables that are part of the human diet. It has been detected in potatoes, soybeans, rice, watermelon, onions, garlic, and beans, with levels ranging from < 1 to ~70 nmol/g of fresh weight [41, 66, 67, 68, 69, 70]. Rapeseed OAS level can even reach 10 μmol/g [71] since OAS accumulation in plants is strongly influenced by sulfur and nitrogen growth conditions. These observations indicate that humans are commonly exposed to OAS through their diet. This raises the possibility that habitual dietary intake may contribute to interindividual variability in circulating OAS concentrations which in turn could be relevant to the observed association between OAS and glycaemia regulation.

This study raises important questions that should be addressed in the near future. First, the mice used in our experiments exhibited only mild glucose intolerance and likely retained largely normal β‐cell function. As a result, we cannot determine whether the impact of OAS on postprandial insulinemia and glycaemia would be more pronounced or attenuated in models of advanced diabetes characterized by severe β‐cell dysfunction and impaired insulin secretion. Second, our experiments were conducted exclusively in male mice, which precludes any conclusions regarding potential sex‐specific differences in the metabolic effects of OAS. Future studies should investigate whether OAS exerts similar glycaemic and hormonal responses in female mice and in models that more closely mimic the pathophysiology of advanced type 2 diabetes. Finally, although OAS is sensitive to basic pH, our pharmacokinetic data demonstrate a very rapid onset in the circulation following oral administration, with plasma levels peaking within 15 min. This suggests efficient absorption in the upper intestine and supports the feasibility of oral delivery using formulations designed for rapid release.

In conclusion, our findings reveal that OAS, an amino acid derivative never studied in mammalian physiology, exerts a significant impact on glucose homeostasis by enhancing glucose‐stimulated insulin secretion and improving postprandial glycaemic control. Even if further investigations are warranted to confirm these effects in humans and to elucidate the molecular mechanisms by which OAS acts on β‐cells, the known roles of amino acids in ATP generation, mitochondrial signaling, mTOR activation, and Ca^2+^‐triggered exocytosis suggest several plausible pathways through which OAS may potentiate glucose‐stimulating insulin secretion. Determining whether OAS engages these canonical amino acid‐responsive mechanisms will be an important direction for future work; nevertheless, our findings already highlight OAS as a promising therapeutic candidate for addressing early impairments in glucose regulation, such as those observed in prediabetes.

## Author Contributions

The authors' responsibilities were as follows: C.B., X.Z., C.D., V.J., and V.D. designed the research; C.B., X.Z., I.H., S.R., S.B., C.D., and V.D. conducted the research; E.L. and S.G.‐D. developed and conducted the chemical analysis of OAS; C.B., X.Z., M.M., C.D., and V.D. analyzed the data; C.B., C.D., and V.D. wrote the manuscript; C.B., S.L., V.J., C.D., and V.D. contributed to the discussion. C.D. and V.D. take full responsibility for the work as a whole, including the study design, access to data, and the decision to submit and publish the manuscript. All authors read and approved the final manuscript.

## Funding

This work was supported by the Prématuration AAP from IDEX Paris‐Saclay, Maturation AAP from SATT Paris‐Saclay, and Prix de Recherche from the Groupe de Réflexion sur l'Obésité et le Surpoids.

## Conflicts of Interest

The authors declare no conflicts of interest.

## Supporting information


**Data S1:** fsb271543‐sup‐0001‐FiguresS1‐S6.pdf.

## Data Availability

The authors have nothing to report.
