# Supplementary material for: O ‐Acetyl‐Serine Supplementation Enhances Insulin Secretion and Improves Postprandial Glycaemia in Lean and Prediabetic Mice
Source: FASEB J. 2026 Feb 20;40(4):e71543. doi: 10.1096/fj.202502925R (PMC12922573; doi:10.1096/fj.202502925R)
Supplement: Supplementary file 1 — Data S1: fsb271543‐sup‐0001‐FiguresS1‐S6.pdf. [file FSB2-40-e71543-s001.pdf]

Supplemental Fig.S1

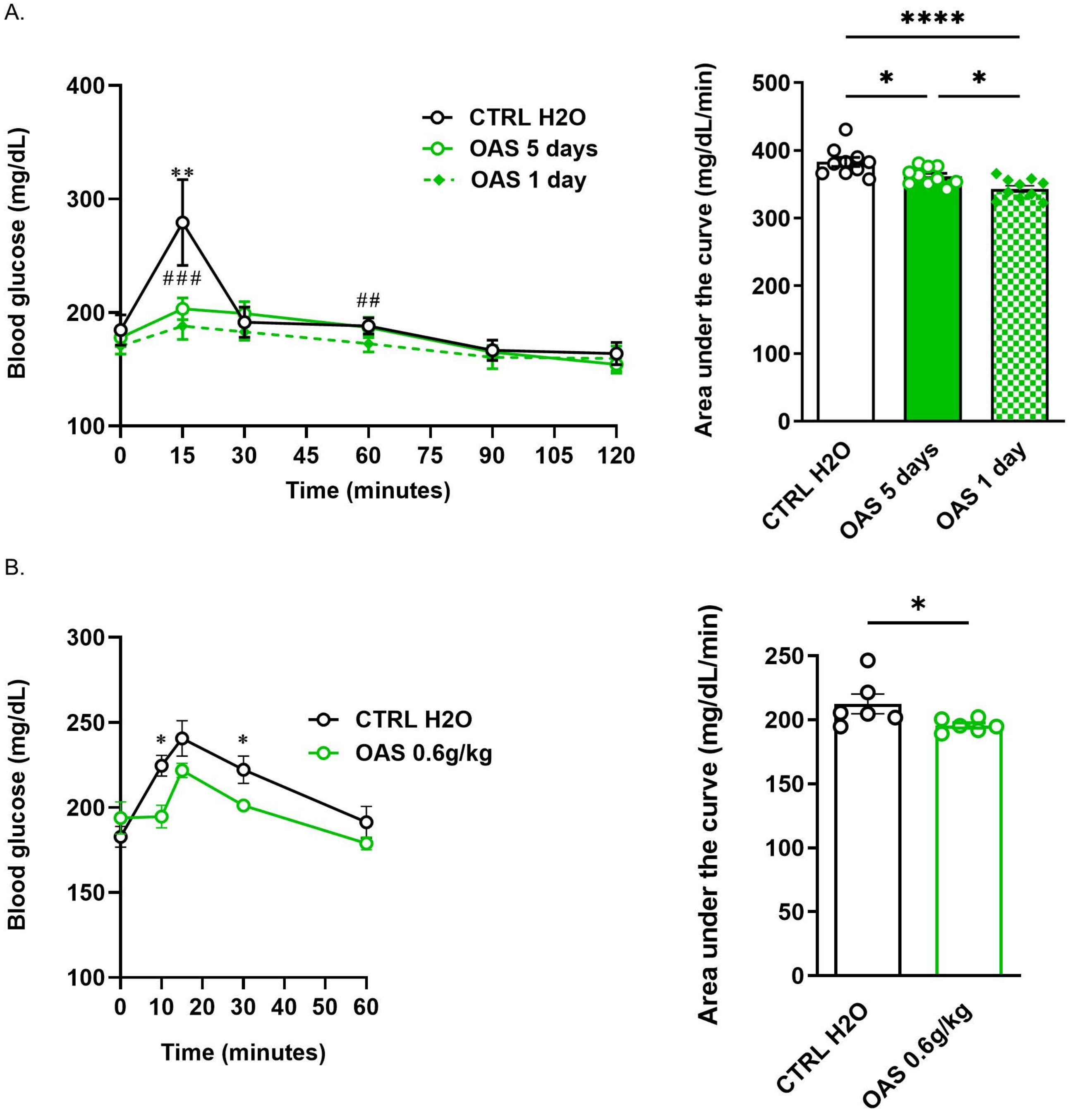

Supplemental Fig.S1:. Acute effect OAS on postprandial glycemia (A) Blood glucose levels during OGTT after 5 or 1 days of oral administration of OAS at 0.6g/kg (n=10). OGTT curves (left panels) and AUC (right panels). OGTT curves and AUC are presented as mean ± SEM. Significance was determined using two-way ANOVA (OGTT curves) and one-way ANOVA (AUCs) with the following p-values: For OGTT curves: * indicates comparisons between OAS 5-days and CTRL H2O; # indicates comparisons between OAS 1-days and CTRL H2O with ** P < 0,01; ### P < 0.0001 and ## P < 0.01. For AUC: *P < 0.05 and **** P < 0.0001. (B) Blood glucose levels during fasting-refeeding test after 1 days of oral administration of OAS at 0.6g/kg or H2O (n=6). OGTT curves and AUC are presented as mean ± SEM. Significance was determined using Wilcoxon paired-test . OAS = *O*-acetyl-serine.

Supplemental Fig.S2

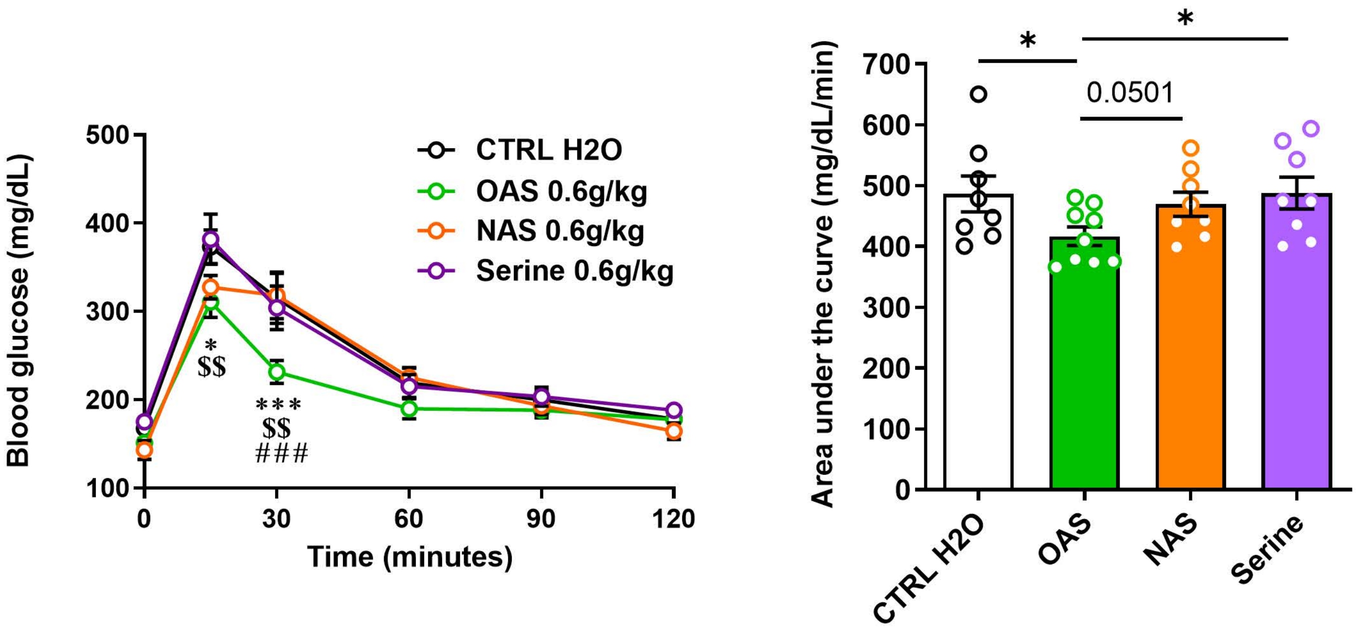

Supplemental Fig.S2:. When OAS is delivered intraperitoneally, its specific effect on glycemia is independent of its metabolites (A) Blood glucose levels during OGTT after 5 days of daily IP administration with H2O, OAS, serine or NAS at 0.6g/kg BW, (n=8-9). OGTT curves (left panels) and AUC (right panels). OGTT curves are presented as mean ± SEM and AUC are presented as mean ± SEM. Significance was determined using two-way ANOVA (OGTT curves) and T-test (AUCs) with the following p-values: For OGTT curves: * indicates comparisons between OAS 0.6g/kg ; CTRL H2O; # indicates comparisons between OAS 0.6g/kg and NAS 0.6g/kg and $ indicates comparisons between OAS 0.6g/kg and serine 0.6g/kg with *P < 0,05; ***P < 0.001; ### P < 0.0001 and $$ P < 0.01. For AUC: *P < 0.05. OAS = *O*-acetyl-serine; OGTT = oral glucose tolerance test; IP = intraperitoneal.

Supplemental Fig.S3

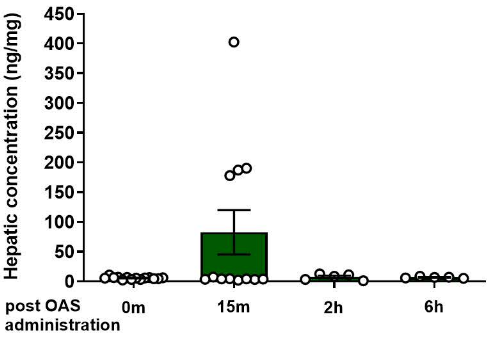

Supplemental Fig.S3: Hepatic OAS concentration. Hepatic levels of OAS after 0, 15 minutes, 2 hours, and 6 hours of a single dose of OAS (1g/kg BW) by gavage, (n=5-14). Data are presented as mean ± SEM. Significance was determined Kruskal-Wallis test. The following p-values were used: . *P < 0.05, **P < 0.01, ***P < 0.005, ***P < 0.0001. OAS = *O*-acetyl-serine.

Supplemental Fig.S4

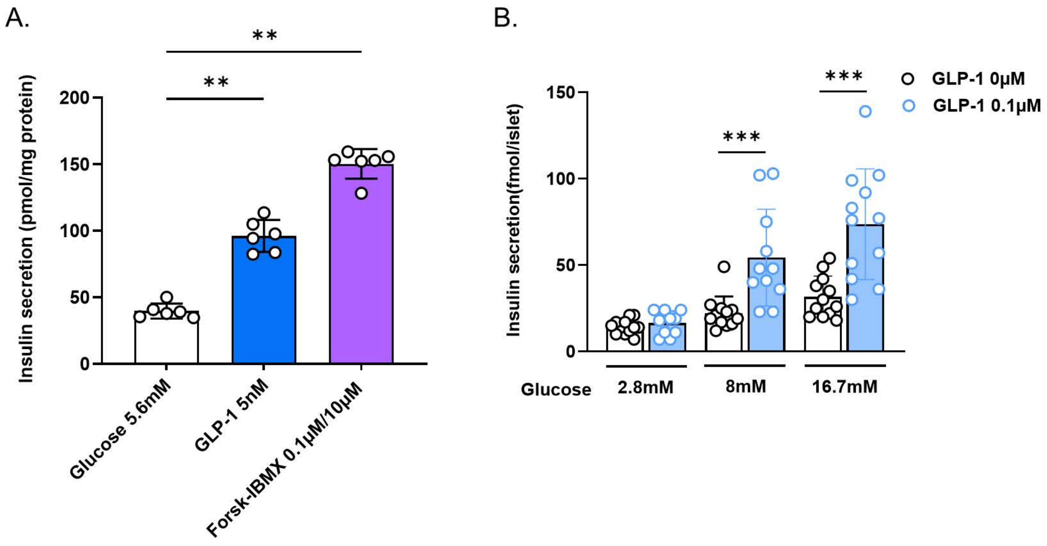

Supplemental Fig.S4: Effect of glucose and references on insulin secretion *in vitro*. (A) Effects of references (GLP-1 and Forskolin/IBMX) on insulin secretion from INS-1 cells after 30 minutes of incubation with glucose (5.6mM) with or without GLP-1 (5nM) or the Forskolin/IBMX mix (0.1µM/10µM) (n=6-14 in two independent experiments). (B) Effects of GLP-1 on insulin secretion from Wistar rat islets after 30 minutes of incubation with glucose (2.8, 8 and 16.7 mM) with or without GLP-1 (0.1µM), (n=10-12). Data are presented as mean ± SEM. Significance was determined using Mann Whitney test. The following p-values were used: *P < 0.05, **P < 0.01, ***P < 0.005, ***P < 0.0001. OAS = *O*-acetyl-serine; GLP-1 = Glucagon-like peptide-1; Forsk = Forskolin: IBMX = 3-isobutyl-1-methylxanthine.

Supplemental Fig.S5

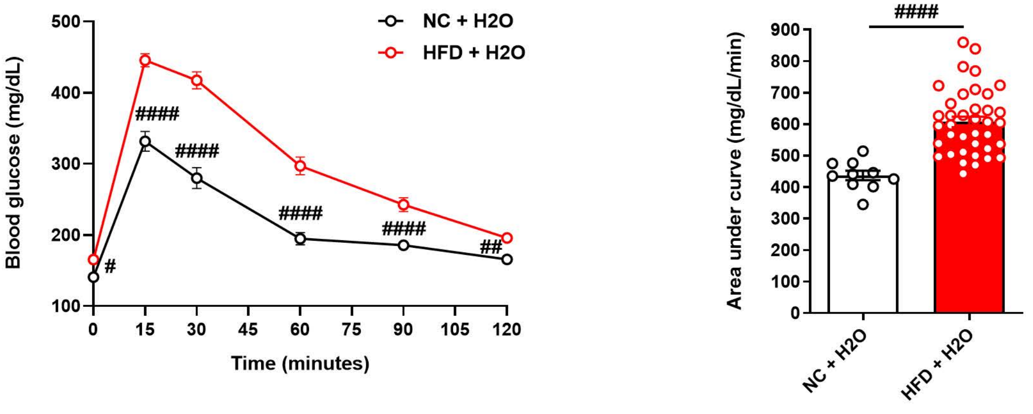

Supplemental Fig.S5: Checking of glucose intolerance establishment. Blood glucose concentrations during OGTT after 2 weeks NC, (n=10) or HFD, (n=38). OGTT curves (left panel) and area under the curve (AUC) of OGTT (right panel). Data are presented as mean ± SEM. Significance at 30 min was determined using two-way ANOVA (OGTT curves) and t-test (OGTT AUC). The following p-values were used: ##P < 0.01; ####P < 0.0001. # indicates the comparison between HFD + H2O and NC + H2O. Normal chow = NC; High-fat Diet = HFD; OGTT = oral glucose tolerance test.

Supplemental Fig.S6

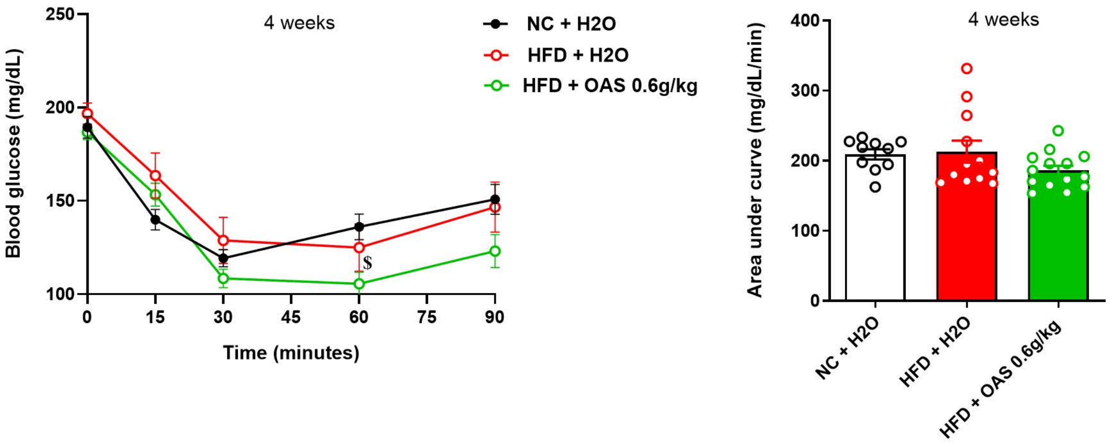

Supplemental Fig.S6: Insulin Tolerance Test. Blood glucose concentrations during intraperitoneal ITT after 4 weeks HFD or NC with two last weeks of treatment by daily gavage with H_2O or OAS at 0.6g/kg BW, (n=10-14). ITT curves (left panel) and area under the curve (AUC) of ITT (right panel). Data are presented as mean ± SEM. Significance was determined using two-way ANOVA (ITT curves) and Kruskal-Wallis (AUC ITT). The following p-values were used: *P < 0.05, **P < 0.01, ***P < 0.005, ***P < 0.0001. * indicates the comparison between HFD + H2O and HFD + OAS; $^{\#}$ to HFD + H2O and NC + H2O; and $^{\$}$ to HFD + OAS vs NC and H_2O. Normal chow = NC; High-fat Diet = HFD; ITT = insulin tolerance test; OAS = *O*-acetyl-serine.
